# Vulnerability of Antioxidant Drug Therapies on Targeting the Nrf2-Trp53-Jdp2 Axis in Controlling Tumorigenesis

**DOI:** 10.3390/cells13191648

**Published:** 2024-10-03

**Authors:** Ying-Chu Lin, Chia-Chen Ku, Kenly Wuputra, Deng-Chyang Wu, Kazunari K. Yokoyama

**Affiliations:** 1 School of Dentistry, College of Dental Medicine, Kaohsiung Medical University, Kaohsiung 807, Taiwan; chulin@cc.kmu.edu.tw; 2 Graduate Institute of Medicine, Kaohsiung Medical University, Kaohsiung 807, Taiwan; r991046@gap.kmu.edu.tw (C.-C.K.); kenlywu@hotmail.com (K.W.); 3 Regenerative Medicine and Cell Therapy Research Center, Kaohsiung Medical University, Kaohsiung 807, Taiwan; 4 Cell Therapy and Research Center, Kaohsiung Medical University Hospital, Kaohsiung 807, Taiwan; 5 Division of Gastroenterology, Department of Internal Medicine, Kaohsiung Medical University Hospital, Kaohsiung 807, Taiwan

**Keywords:** antioxidation, heterogeneity, reactive oxygen species, redox homeostasis, cancer therapy, tumor suppressor p53

## Abstract

Control of oxidation/antioxidation homeostasis is important for cellular protective functions, and disruption of the antioxidation balance by exogenous and endogenous ligands can lead to profound pathological consequences of cancerous commitment within cells. Although cancers are sensitive to antioxidation drugs, these drugs are sometimes associated with problems including tumor resistance or dose-limiting toxicity in host animals and patients. These problems are often caused by the imbalance between the levels of oxidative stress-induced reactive oxygen species (ROS) and the redox efficacy of antioxidants. Increased ROS levels, because of abnormal function, including metabolic abnormality and signaling aberrations, can promote tumorigenesis and the progression of malignancy, which are generated by genome mutations and activation of proto-oncogene signaling. This hypothesis is supported by various experiments showing that the balance of oxidative stress and redox control is important for cancer therapy. Although many antioxidant drugs exhibit therapeutic potential, there is a heterogeneity of antioxidation functions, including cell growth, cell survival, invasion abilities, and tumor formation, as well as the expression of marker genes including tumor suppressor proteins, cell cycle regulators, nuclear factor erythroid 2-related factor 2, and Jun dimerization protein 2; their effectiveness in cancer remains unproven. Here, we summarize the rationale for the use of antioxidative drugs in preclinical and clinical antioxidant therapy of cancer, and recent advances in this area using cancer cells and their organoids, including the targeting of ROS homeostasis.

## 1. Introduction

**Figure 1:**
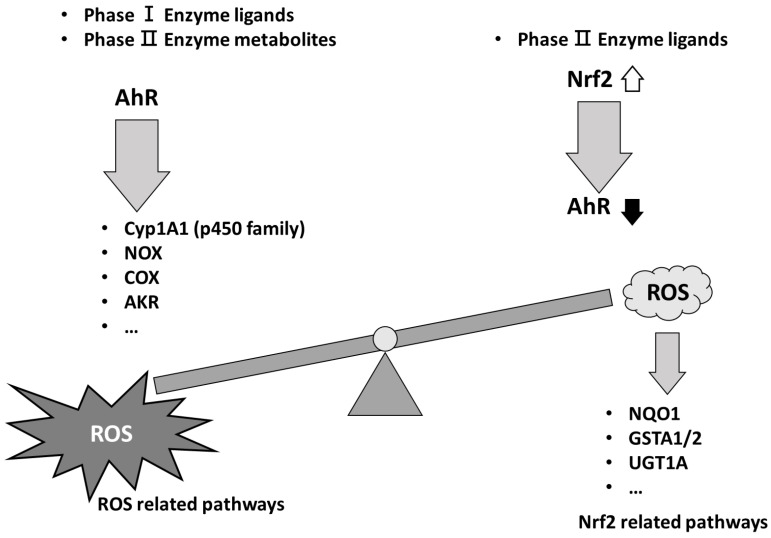
Schematic model of ROS balance induced by phase I enzyme ligands and phase II enzyme ligands. Phase I enzyme ligands or phase II enzyme metabolites are able to induce the AhR activation, to generate ROS production. By contrast, the phase II enzyme ligands induce Nrf2 activation to decrease the level of AhR and ROS production, which results in the phase II enzyme metabolites. Thus, ROS balance is controlled by AhR and Nrf2. Cyp1A1 (p450 family); cytochrome p450 family Cyp1A1, NOX; nitrogen oxides, COX; cyclooxygenase, AKR; aldo-keto reductase, NQO1; NAD(P)H dehydrogenase (quinone) family, GSTA1/2; glutathione S transferase alpha 1 and 2, UGT1A; UDP glucuronosyltransferase family 1 member A complex locus. Black arrow indicated the decreased level of AhR and white arrow indicated the increased level of Nrf2 expression.

It is known that the control of balance between reactive oxygen species (ROS) and redox status is an important way to address the occurrence and development of tumor cells without compromising normal cells [1]. This idea depends upon the theory that cancers have a homeostatic balance of oxidation, which is produced by phase I enzyme ligands, and antioxidation, which is mediated by phase II enzyme ligands and is arranged to favor the hallmark actions of normal cells or cancer cells, such as proliferation, survival, migration, and metastasis (Figure 1) [2]. Thus, the ROS balance is critical for regulating oxidative stress and antioxidation and mediated by transcription factors such as the aryl hydrocarbon receptor (AHR) and nuclear factor erythroid 2-related factor 2 (NRF2), respectively. The extensive redox conditions damage biocomponents, and cancers are known to compensate for this damage by enhancing the expression of antioxidants. Thus, the development of cancers depends on these antioxidant proteins to regulate the oxidation and antioxidation status of macromolecules within cells to maintain the ROS balance. A switch in cancer redox activity produced by antioxidation or increased ROS production causes the threshold of the ROS balance of cancer cells to be altered, which causes cell cycle arrest or cell death [3]. Redox-modulating drugs that cause apoptosis are important in defining ROS-induced cell death, which is important for anticancer therapy [3]. AHR and NRF2 are critical factors for protection against environmental stresses. The phase I enzyme system facilitates the oxidative reduction of xenobiotics, which converts intermediate metabolites, including electrophiles. The phase II enzyme system involves conjugating a hydrophilic moiety (i.e., glucuronate, sulfate, glutathione, or glycine) to phase I metabolites. This reaction involves enzymes, such as transferases, and the phase I metabolites are subsequently transformed into water-soluble molecules. Thus, these metabolites or AHR-induced ROS can trigger the phase II enzyme system to reduce the endogenous ROS using the NRF2-KEAP1 (Kelch-like ECH associated protein 1) system to maintain the endogenous ROS balance within the cells. AHR controls phase I enzymes, including the CYP450 family (i.e., CYP1A1, CYP1A2, and CYP1B1), nitrogen oxides (NOXs), cyclooxygenase (COX), and aldo-keto reductase (AKR), while NRF2 controls phase II enzymes, such as glutathione S-transferase α1 (GSTA1), glutathione S-transferase π1 (GSTP1), and the uridine diphosphate glucuronosyltransferase family (UGTs). The interaction between the AHR and NRF2 pathways is essential for cellular protection against the toxic byproducts of AHR. The oxidative reactive species (ROS) are toxic byproducts of aerobic metabolism that are harmful to cells and cause oxidative damage to DNA, proteins, and lipids, which might lead to cell death. ROS production is compensated by reversible reactions, such as antioxidation, to maintain a balance of ROS levels. An elevated level of ROS is detrimental to the control of oxygen toxicity, whereas physiologically low levels of ROS induced by antioxidative mechanisms serve as intracellular signaling mechanisms. Thus, the endogenous control of ROS homeostasis is a cellular defense mechanism against toxicants and oxidative stress (Figure 1).

Although drugs that control ROS are expected to have therapeutic applications in cancer, drugs targeting ROS have shown only limited success in preclinical trials compared with other anticancer drugs [4]. These results raise the possibility that tumor cells may not be more sensitive to ROS than normal cells. Thus, these trials produced less success than anticipated. The lack of biomarkers to measure endogenous redox levels might also prevent success [5]. Furthermore, the complexity and redundancy of drugs targeting ROS/redox are not well characterized. Recent reports have shown the heterogeneity of phase II enzyme reagents against cancer organoids [6]. Thus, the use of antioxidants for cancer treatment should be approached with caution. In this review, the heterogeneity of phase II enzyme agents is discussed in the context of cancer treatment and how to avoid these difficulties in preclinical trials of phase II drugs for cancer treatment.

## 2. ROS in Cancer Cells

Assuming a crucial role for ROS in cancer cells, controlling endogenous ROS by altering the function of mitochondrial constituents is a possible anticancer strategy. These strategies may inhibit ROS-mediated cancer occurrence and progression by causing oxidative damage, such as ROS-mediated apoptosis [7,8]. Thus, preclinical research on antioxidant production and weak pro-oxidants was conducted to understand their merits. Compared with normal cells, cancer cells generate excessive ROS, increasing their sensitivity to further increases in ROS-related cell damage and committing them to apoptosis. Pro-oxidants may thus have antitumor functions. Therapeutic trials of antioxidants targeting ROS control have included nonenzymatic drugs, such as NRF2 agonists [9] and vitamins [10,11], or targeting ROS via the enzymatic production of antioxidants, such as nitrogen oxide inhibitors [12,13], superoxide dismutase mimetics [14], *N*-acetylcysteine, and glutathione (GSH) esters [15,16]. Many such pre and clinical trials have been conducted (Table 1), and many such agents against ROS production have been developed, but their actions involve ROS homeostasis and antioxidation, which are not specific to cancer.

For example, the repression of glutathione peroxidase 2 (GPx2) in breast cancer may enhance cancer progression due to hypoxic signals, aberrant vascularization, and a metabolic switch to the aerobic glycolysis/oxidative phosphorylation (OXPHOS) axis. The complex expression of GPx2 depends on metabolic-specific drives and microenvironments, such as hyperproliferation-derived hypoxia.

Another consideration is that ROS have a dual function in cancer occurrence, which might depend on endogenous ROS (Figure 1) [17].

**Table 1:** The variation of redox levels in different cancer cells. Various effects of antioxidant functions are also shown.

Tumor Type	Redox Level	Effects	References
Urinary tract & bladder	↓	GPx2 ↓ Poor prognosis patient samples	[18]
Esophageal carcinoma	↓	GPx2 ↓ Poor prognosis patient samples	[19]
Early-stage of lung adeno carcinoma (LuAD)	↓	GPx2, ALDH3A, GLRX, PPK ↓ Increase ROS dependent caspase activity Aggressive phenotype in early stage of hypoxic tumor	[20]
T1 Bladder carcinoma	↓	pT1 stage GPx2 ↓ Early-phase invasion	[21]
Breast cancer	↓	GPx2 ↓ Tumor heterogeneity Metabolic plasticity Malignant progression ROS/HIF1/VEGFA pathways activation	[22]
Pancreas cancer	↓	Proliferation ↓ Invasion ↓ Metastasis ↓	[23]
Hepatocellular carcinoma	↑	Poor prognosis	[24]
Gastric carcinoma	↑	Markers of primary tumor and metastatic form	[25]
Lung carcinoma	↑	Tumor initiation Cisplatin resistance	[26]
Lung adenocarcinoma	↑	Tumor formation ↑ Independent prognostic factor of recurrence free survival (RFS)	[27]
Non-small cell lung adenocarcinoma	↑	Gpx2 ↑: as an oncogene by inhibit of apoptosis, promoting MET, glc uptakes BMP ↓: as an Tumor suppressor via inducing apoptosis cell cycle arrest	[28]
Prostate cancer	↑	PC2, RFS prognostic model ↑ Wnt/β-catenin/EMT pathway ↑	[29]
kRas mutated lung cancer cells	↑	Malignant progression Cisplatin resistance	[30]
Colon cancer	↑	Microsatellite instability ↑	[31]
Gastric cancer	↑	Patient survival, Tumor progression in ROS-KYNU-KYN-AhR signal in GPx2 knock down cells	[32]
NSCLC (Non-small cell lung cancer)	↑	EMT metastasis of NSCLC by PI3K/Akt/mTOR/snail axis to remove ROS Prognosis is OK	[33]
NSCLC	↑	Breast cancer metastasis suppressor 1 like (BRMS1L) ↓: is associate with cancer growth Gpx2 mediated oxidative stress repair ↓ BRMS1L knockdown characterized NSCLC cells to ROS induce	[34]

Excessive ROS production leads to a defective antioxidant defense mechanism and thereby impairs the balance between antioxidants and pro-oxidants. Recent reports revealed the dichotomous nature of ROS in tumor cells, depending on the stage of cancer progression (early stage or late stage) or differences in endogenous ROS levels. Elevated ROS production in cancer cells initiates adaptation, which maintains the ROS balance by antioxidation reactions. In precancerous stages or early stages of cancer progression, moderate ROS levels induced tumorigenesis, tumor promulgation, metastasis, and survival [35]. With tumor progression, ROS levels elevated beyond a toxic threshold lead to cell death, apoptosis, and senescence [36,37].

This dichotomy further complicates the antioxidation response to the decomposition of H_2_O_2_ through a change in the glutathione-SH (GSH)–glutathione sulfide (GSSG) axis. Hypoxia-inducible factor-1β, also known as AHR nuclear translocator (ARNT), is a component of the complex of phase I enzymes with AHR. Thus, crosstalk between AHR signals and hypoxia signals might occur, leading to overlap. In addition, the development of cancers also involves contrasting levels of GPx2 expression, which are correlated with the progression of cancer and poor prognosis. Decreased GPx2 expression was detected in colon cancer, pancreatic cancer, cancers of the bladder and urinary tract, and esophageal colon carcinomas, whereas increased GPx2 expression was detected in several carcinomas [38]. This apparent contradiction of GPx2 levels might be due to differences in the levels of the intact endogenous ROS production machinery [39].

## 3. NRF2 Has Dual Roles in Tumorigenesis

NRF2 has dual and contradictory roles in cancer [39]. Abnormal increased expression of NRF2 can be related to a poor prognosis. The constitutive level of NRF2 in many cancer cells might induce the expression of pro-survival genes and promote the proliferation of tumor cells via metabolic alterations, inhibit cancer specific-apoptosis, and increase the self-renewal function of cancer stem cells. Moreover, NRF2 contributes to chemoresistance, radio-resistance, and inflammation, inducing carcinogenesis. Many NRF2 inhibitors have been reported to treat cancers [40]. These trials targeting NRF2 might provide a new tool in cancer treatment.

### 3.1. Tumor Suppressive Actions of NRF2

The NRF2 protein is activated by tumor suppressor gene products such as breast cancer susceptibility gene I and p21^Cip1^ by inhibiting KEAP1/NRF2 complex formation in microenvironmental cells in tumors [41,42]. The degradation of the oncogene tyrosine kinase Fyn blocks its activation [43]. Nrf2-deficient mice reduced the production of antioxidants and repressed the expression of markers specific for phase II enzyme-encoded genes. Quinone induced severe oxidative stress rendered Nrf2-deficient mice more prone to skin cancer, while the Nrf2-mediated expressions of NAD(P)H quinone dehydrogenase 1 (Nqo1) and Gst were inhibited [44]. Similarly, Nrf2-deficient mice exposed to carcinogens developed more cancers, including stomach [45], liver [46], and urinary bladder [47], as compared with wild type mice, suggesting that Nrf2 is the critical factor controlling inflammation.

Many compounds isolated from plants, including carnosol, curcumin, resveratrol, and sulforaphane, and synthetic chemicals like oltipraz and oleanane triterpenoids, exhibit chemo-preventive functions by activating the Nrf2-antioxidant response element (ARE)-regulated genes. These Nrf2 activators mainly control the antioxidation reaction by changing the intermolecular disulfide (S–S) bonds between two cystine residues of Keap1 proteins at Cys273 and Cys288 to commit the accumulation of Nrf2 [48]. Sulforaphane (SFN) is committed to inducing the transcription of phase II enzyme-encoding genes while inhibiting phase I enzyme-encoding genes and facilitating the cell death of cancer cells through the TP53 mechanism [49]. In addition, SFN targeted nuclear factor kappa B (NF-κB) [50] and activation protein-1 (AP-1) complexes with an anti-inflammatory effect [51]. Other synthetic oleanane triterpenoids were found to inhibit tumorigenesis by inhibiting the transcription of oncogenes, including k-*RAS*, *TP53*, *BRCA1*, and Erb-B2 receptor tyrosine kinase 2, in some cancers [52,53].

A lot of therapeutics that control NRF2 activity have been currently applied in clinical trials; these drugs play a significant role in repressing cancers and could be augmented further by chemo-preventive compounds. Paradoxically, NRF2-deficient cancer cells were found to be more prone to the death of cancerous cells by oxidative stress but more resistant to chemo-preventive compounds. Thus, effective drugs targeting NRF2 pathways represent a crucial strategy to develop useful chemo-preventive medications. However, we need to accumulate further pre-clinical data to prove the efficiency of these drugs against cancer in human patients.

In Nrf2-knockout mice, the level of nitric oxide synthase activated by cyclooxygenase 2 and the level of tumor necrosis factor (Tnf) are greater than those in normal mice, indicating that Nrf2 is able to suppress the proinflammatory molecules [54]. In addition, Nrf2-dependent activation of Nqo1 decreased the levels of Tnf and interleukin 1 after incubation with lipopolysaccharide. Although ROS are the molecular targets of NRF2-mediated anti-inflammatory effects, NRF2 might function as an anti-inflammation mediator without inducing ROS, which was reported by regulating the expressions of the axis encoding the macrophage receptor with collagenous structure and CD36, which are not concerned with the oxidative response [55]. Furthermore, NRF2 protected against cell damage induced by H_2_O_2_ exposure via the p38/MAPK pathway [56,57]. Similarly, NRF2 inhibited the NF-κB pathway by stabilizing the inhibitor of nuclear factor κB kinase subunit-α (IKK-α) to inhibit the degradation of IKK-β [58]. By contrast, the NF-κB p65 subunit was reported to compete with NRF2 through the CH-KIK domain of the cyclic AMP response element binding protein (CREB) binding protein CBP coactivator [59]. Thus, these different mechanistic inputs of NRF2 should be clarified to determine how it suppresses oncogenicity by controlling the level of ROS within cells.

### 3.2. Oncogenic Functions of NRF2

NRF2 is usually constitutively activated in tumorigenic cells. For example, somatic mutations in *KEAP1* and/or *NRF2* genes, exon skipping in the *NRF2* genome, methylation/demethylation of the *KEAP1* promoter, accumulation of p62/sequestosome-1, and mutation of fumarate hydrolase have been reported [60,61] Constitutively activated NRF2 promoted cell proliferation through metabolic changes; inhibiting apoptosis; promoting angiogenesis, and invasion/metastasis; and promoting drug resistance in various cancers has also been reported [62,63]. Forced expression of NRF2 promoted the transcriptions of the oncogenes *MYC*, *KRAS*, and *BRAF* [64]. Moreover, NRF2 activated oncogenes by suppressing the activity of tumor suppressor gene products like phosphatase tensin homolog/glycogen synthetase kinase 3/β-transduction repeat-containing E3 ubiquitin-protein ligase [63]. NRF2 increased the proliferation of tumor cells by increasing the expression of glycolytic enzymes including glucose-6-phosphate dehydrogenase, phosphoglucomutase dehydrogenase, transketolase and trans-aldolase [64], which control genes involved in fatty acid-lipid metabolism [65], growth-associated genes [66], and cell cycle regulators [67]. The activation of NRF2 via the glucose-regulated protein 78/phosphorylated protein kinase RNA-like ER kinase/NRF2 signaling pathway enhanced the transcriptions of glycolytic genes and simultaneously induced the inhibition of the TCA cycle related genes, which enhanced the Warburg effect [68].

Increased levels of NRF2 suppressed the endogenous ROS level in cancer stem cells (CSCs) compared with noncancer stem cells, suggesting the enrichment of stemness phenotypes [69,70,71,72]. For example, CSCs with reduced mitochondrial-derived ROS exhibited cancer stemness-associated properties, such as epithelial-to-mesenchymal transition (EMT) [68,73]. Similarly, persistent activation of NRF2 improved the self-renewal ability of CSCs by maintaining cell quiescence and reducing the intracellular levels of ROS [74,75]. Mesenchymal stem cells (MSCs) in the microenvironment cells promoted the spread of cancer cells by enhancing their motility and invasion ability [76,77]. Thus, NRF2 was required to maintain the stemness of MSCs and causing their cell deaths under an oxidative stress condition [78]. Furthermore, because the continuous expression of NRF2 significantly benefited the cancer cells, these cells frequently developed NRF2 dependency [79,80].

In addition, NRF2 -derived oncogenic activity also stimulated angiogenesis capacity, mainly by activating heme oxygenase 1 (HO-1) [81], which in turn stimulated the vascular endothelial growth factor to enhance angiogenesis [82]. siRNA against NRF2 downregulated HO-1 and sensitized acute myeloid leukemia cells to TNF-induced cell death. This effect indicates that NRF2 inhibits the apoptosis of cancer cells by regulating HO-1 [83]. In addition, NRF2 upregulated the antiapoptotic protein B-cell lymphoma 2 (Bcl2), while it downregulated the activity of BAX and caspase 3 to protect against etoposide/radiation-mediated cell death following drug resistance [84]. Furthermore, NRF2 inhibited the activation of proapoptotic JNKs [85] and induced selective autophagy reactions via Keap1 degradation [86,87]. However, the forced expression of NRF2 allowed autophagy-dependent cancer cells to overcome the loss of autophagy function [88].

Increased accumulation of NRF2 in the nucleus was associated with proliferation capacity. For instance, phosphoinositide 3-kinase (PI3K)-Akt activation coupled with depletion of Keap1 resulted in Nrf2-dependent proliferation of hepatocytes and cholangiocytes [89,90]. However, because the ability of stable Nrf2 was not sufficient to alter it from a cancer defender to a cancer driver, the additional mutations of oncogenes and tumor suppressor genes were essential [91,92,93]. Keap1 mutations, such as Kras/Hras mutations and Trp53 loss of function, are required to produce NRF2-dependent cancer models [94]. Furthermore, NRF2-dependent malignancies with somatic mutations of KEAP1 and NRF2 differ depending on the specific organs. Thus, tissue-specific variation is another issue for the study of NRF2-dependent cancer, which should be discussed further.

In resistance to medical therapy, NRF2-controlled drug efflux transporters are predictors of resistance for drug inoculations. For example, multidrug resistance protein 1 (MDR1), multidrug resistance-associated proteins 1-5 (MRP1-5), and breast cancer-resistant protein (BCRP) are critical for controlling issues to regulate the therapy because they induced the abnormal NRF2 activation to induce the widespread chemoresistance [95,96,97,98]. These issues might comprise complementary trials for drug delivery to cancer cells.

## 4. NRF2-Targetted Drugs in Cancer Prevention

**Figure 2:**
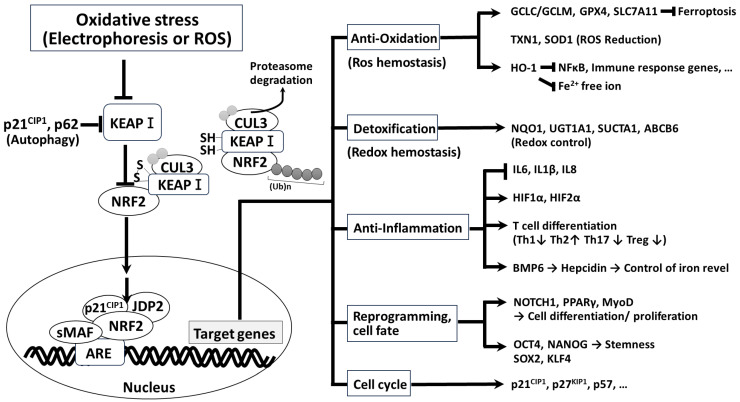
Schematic representation of the actin mechanism of the NRF2 signaling pathway. Under basal conditions, a KEAP1/CUL3/RBX1 complex binds to NRF2 and promotes ubiquitination to commit proteasomal degradation. Upon activation of NRF2 by electrophiles or reactive oxygen species (ROS) and other ligands, NRF2 enters the nucleus to associate with sMAF, p21^Cip1^, JDP2, binds to ARE cis-element, and modulates the expression of several gene families such as anti-oxidation, detoxification, anti-inflammation, reprogramming and cell fate decision, and cell cycle. GCLC (glutamate-cysteine ligase catalytic subunit), GCLM (glutamate-cysteine ligase modifier subunit), GPX4 (glutathione peroxidase 4), SLC7A11 (solute carrier family 7 member 11), TXN1 (thioredoxin1), SDO1 (super oxide dismutase type 1), HO-1 (heme oxygenase 1), NQO1(NAD(P)H quinone dehydrogenase 1), UGT1A1 (UDP glucuronosyltransferase family 1 member A1), SULTA1 (Sulfotransferase family 1A member 1), ABCB6 (ATP-binding cassette superfamily B member 6), IL (interleukin), HIF 1α (hypoxia inducible factor 1α), BMP6 (bone morphogenic protein 6), NOTCH 1 (neurogenic locus notch homolog protein 1), PPARγ (peroxisome proliferator-activated receptor gamma), and MyoD1(myogenic differentiation 1).The circle indicated the transcriptional event in the nucleus.

In the initiation stage of cancer, NRF2 activation can suppress carcinogenesis. Under normal conditions, NRF2 maintains antioxidation homeostasis and exerts anti-inflammatory effects and antitumorigenic effects, thus supporting cell survival (Figure 2). NRF2 can control the transcription of phase II enzyme genes and activate a series of defective mechanisms through the Keap1/Nrf2/ARE axis, maintaining cellular redox homeostasis. NRF2′s anti-inflammatory and anticancer activities avoid cell damage, thus benefiting the survival of normal cells. In this respect, NRF2 activation is critical for cancer prevention. By contrast, the overactivation of NRF2 also protects cancer cells and promotes their growth. Excessive NRF2 signaling plays a tumor-promoting role, mainly by maintaining the proliferation signals, infinite replication, continuous angiogenesis, resistance to apoptosis and ferroptosis escaped from immune destruction, and promotion of invasion and migration [43,48].

### Heterogeneity of Antioxidation Drugs against Cancer

Wu et al. (2023) recently reported the heterogeneity of redox ligands controlling the growth and progression of human gastric cancer organoids [6]. They performed experiments to compare the efficiency of three drugs, perillaldehyde (PEA) and cinnamaldehyde (CE), and the regular antioxidative drug sulforaphane (1-isothiocyanato-4-(methane sulfinyl) butane; SFN), and measured cancer occurrence by assessing xenotransplantation in SCID mice. PEA extracted from *Perilla frutescens* [99] showed antifungal, and antioxidant activities in addition to other biological functions [100,101,102]. PEA activates NRF2 and represses oxidative stress-induced innate immunity in human keratinocytes [103]. CIN is a β-unsaturated aldehyde from cinnamon [104]. In addition, the antioxidant activity of CIN was demonstrated in mice [105]. Exposure to CIN-induced autophagy-dependent cell death through epigenetic alteration by the histone methylating enzyme G9a in the endoplasmic reticulum [106]. CIN inhibited the AHR axis and induced an NRF2-mediated antioxidation response [107]. Thus, PEA and CIN might function in NRF2-dependent antioxidation to inhibit ROS generation without affecting AHR-mediated oxidative stress pathway. SFN, as a dietary isothiocyanate synthesized from a precursor in *Brassica*, was also examined. It is one of the most typical ligands of phase II enzymes pathway, including NQO1, GST α-1, and HO-1, which are required to eliminate chemically damaged DNA. SFN induced cell cycle arrest at S-phase and apoptosis in a TP53-dependent manner in gastric cancer (GC) cells [108]. In addition, SFN inhibited the metastasis of breast cancer [109].

**Figure 3:**
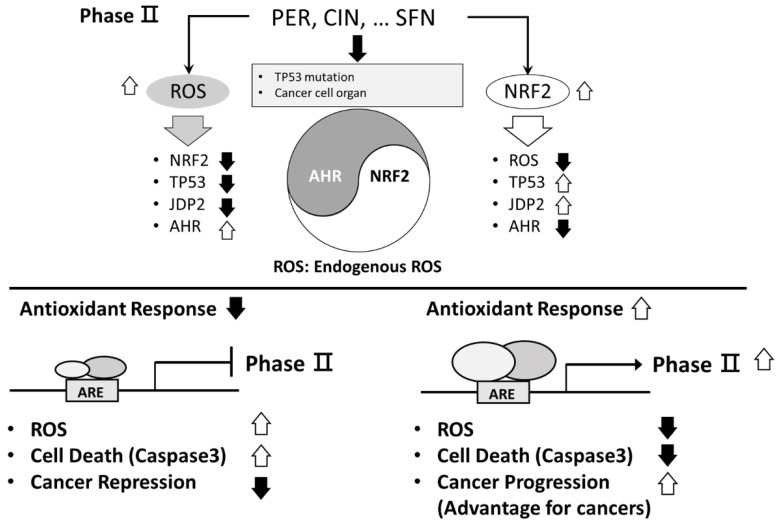
Divergent functions of PER, CIN, and SNF to control gene expressions of AHR and NRF2. The heterogeneous function of phase II enzyme ligands to affect the ROS level and Nrf2 gene expression is illustrated. The p53 mutation status and the normal and cancer cells context are critical for the bifunctional effects of antioxidation drugs to control the balance of ROS. Thus, the endogenous ROS in the cells we targeted should be examined. Higher ROS levels decreased the expression levels of Nrf2, p53, and Jdp2, and lower ROS decreased the generation of ROS and increased p53 and Jdp2 levels. The ROS level was controlled by the AhR expression, and antioxidation was controlled by Nrf2-mediated responses. PER; perillaldehyde, CIN; cinnamon aldehyde, SFN; sulforaphane, ARE; antioxidative response element. The white arrows indicated the increased levels of genes or functions, and the black arrows indicated the decreased levels of genes or functions.

Another issue is the differential outputs on function of AHR- and NRF2-mediated phase I and II pathways. Both PEA and CIN induced an NRF2-mediated phase II enzyme response, which was mediated by ARE-containing genes [109] but was not involved in the phase I pathway [107]. By contrast, the effects of SFN were reported to be involved in both enzymatic pathways; SFN produced NRF2-dependent phase II promoter activation and AHR-dependent activation of phase II promoters such as NQO1 [110,111]. This activation provides unambiguous evidence of the different actions of PEA/CIN and SFN in preventing cancer-inducing activity. It is surprising to observe the contrasting actions of PEA/CIN and SFN in inhibiting gastric cancer development, ROS generation, and cellular apoptosis. TP53 mutation might explain these differential effects because 3-D organoids exhibited TP53 mutations, which might alter the phase I and phase II enzyme reactions (Figure 3).

## 5. Phase I Drugs in Clinical Trials

Phase I and II enzyme pathways are known to overlap and are regulated by the “ARF–NRF2” gene battery [112]. In addition, the AP1/ATF transcription factor and histone chaperone Jun dimerization protein 2 (Jdp2) were found to play a role in regulating AHR promoter activity via the NRF2 complex [113]. This mechanism was named the AHR–NRF2–JDP2 gene battery.

In the case of AHR therapeutics, the present application of AHR antagonists in cancer disorders is a reasonable strategy for preclinical trials. However, although many trials of AHR-targeted drugs and NRF2-target genes for cancer or disease have been conducted, most of them did not result in completed FDA approvals. The current trials are listed (Table 2) [114,115]. AHR antagonists inhibit immune suppression, and AHR agonists overcome chronic inflammation and autoimmune disorders in cancer patients. The AHR antagonist IK-175 is well known to be effective in an anticancer therapeutic trial when combined with anti-PD1 antibodies. A phase Ia/b open-label study of OK175, alone or in combination with nivolumab, which suppressed locally advanced solid tumors and urothelial carcinoma, has been reported (NCT04200963) [34]. The microbial metabolite indole-3-aldehyde (3-IAld) exhibited strong anti-inflammatory activity via AHR [116,117]. Compelling results with 3-IAld were obtained in a dextran sodium sulfate (DSS)-mediated inflammatory bowel disease of mouse model. 3-IAld repaired colon damage and improved epithelial barrier integrity through AHR, and IL22 cured immune checkpoint inhibitor (ICI)-induced colitis but did not block the antitumor effect of ICI [118]. These data indicate that the AHR ligand of 3-IAld could be developed as an agent for treating human disease. 

BAY 2416964 is a novel oral AhR inhibitor that prevented AHR ligand-induced immunosuppressive effects and enhanced the proinflammatory activity of antigen-in-human phase I clinical trials [119]. Studies in vitro showed that BAY 2416964 restored immunological activity in human and mouse cells, stimulated antigen-specific cytotoxic T-cell responses, and killed tumors. Studies in vivo showed that its oral application was well tolerated and demonstrated antitumor activity in a syngeneic mouse cancer model.

## 6. Phase II Drugs in Clinical Trials

Clinical current trials using phase II enzyme drugs have been reported (Table 2) [124,148,149,150,151].

For example, SFN has achieved only limited success in prostate cancer patients [152]. However, SFN is not effective for treating breast cancer patients [153]. SFN inhibited the progression of GC [108,154,155,156,157]. Thus, further studies are required to determine the usefulness of SFN.

In addition, the mutation status of oncogenes and antioncogenes is also crucial for the efficacy of treatment with phase II enzyme drugs. The expressions of TP53, NRF2, and JDP2 were tremendously repressed in the growth of organoids exposed to PEA and CIN as compared with those in control normal organoids. However, a 1.5- to 1.75-fold increase in NRF2 and TP53 expression was found in SFN-treated organoids compared with control organoids. Exposure to PEA or CIN repressed the expression of proteins of the TP53–NRF2 axis and a third factor histone chaperone JDP2, but SFN increased the expression of TP53 and NRF2 proteins. In the case of a tumor, the p53 mutation itself did not induce the tumor formation, but tumors developing from areas with p53 mutation and loss of heterozygosity were larger and demonstrated extensive chromosomal instability compared with lesions arising in normal epithelium [158]. Most TP53 mutations in cancers are found as missense mutations rather than truncations or deletions. Both dominant-negative effects and gain-of-function activities were observed in the case of mutant p53 [159]. Mutant TP53 interacts with NRF2, but both positive and negative effects of NRF2 have been reported [160,161,162]. Non-small cell lung cancers carrying mutant TP53 but not mutant NRF2 or KEAPl displayed higher levels of NRF2 mRNA than wild-type TP53 tumors [163]. Similarly, the oncogenes *KRAS*, *BRAF*, and *MYC* promoted the increased transcription of *NRF2* gene and its target genes, which might induce a greater decrease in cellular molecules [62]. Mutant p53 prolonged TNFα-induced NF-κB signaling [164], and mutation of p53 can upregulate Nrf2 via the NF-κB axis. However, in 3-D gastric cancer organoids, PEA and CIN treatment reduced the protein expression of TP53 and Nrf2 and reduced ROS and caspase 3 activity [6].

By contrast, SFN exposure induced NRF2 and other redox functions to be dominant. Some reports have indicated that the levels of phase II enzymes, such as NQO1, are increased in cancer tissues compared with healthy tissues and that NQO1 stabilized the wild-type TP53, especially under oxidative stress [165]. Compared with those of wild-type TP53, the TP53 mutants exhibited increased binding to NQO1 [166]. By contrast, the function of NRF2 as an antioxidation response factor was blocked in the case of R273H in p53-expressing cancer cells upon oxidative stress, and the NRF2 antioxidant response was impaired because of the decreased expression of NQO1 and HO-1 [167].

Mutant p53 enhanced the Warburg effect by activating the glucose transporters GLUT5/6 and GLUT3 via the NF-κB axis and GLUT1 by stimulating its transfer to the plasma membrane. Various glycolytic enzymes are also stimulated by mutant p53, which caused increased glucose uptake and glycolysis [168]. The effect of the microenvironment on p53 mutations can also affect cancer generation.

One TP53 mutant, APR-246, has been tested in clinical applications [169]. The APR-246 mutant inhibited thioredoxin reductase 1 (TrxR1) [170], thioredoxin (Trx), glutaredoxin, and ribonucleotide reductase [171] and depleted cellular GSH [172,173]. Recent studies revealed that APR-246 was not specific to cancer cells, but its effects might be cell-type specific [174].

In general, the expression of p53 counteracted the expression of ARE-containing antioxidant genes such as cystine/glutamate transporter (SLC7a11; x-CT), NQO1, and GST-α1, which are Nrf2 targets [175]. p53 deletion increased ROS, DNA oxidation, and mutations. The introduction of dietary supplementation with the antioxidant *N*-acetylcysteine subsequently improved karyotype stability and prevented the early onset of mutations for tumorigenesis. Three p53 mutations, K117R, K161R, and K162R, were generated and resulted in impaired p53-mediated cell cycle arrest, senescence, and apoptosis. Unlike p53-knockout mice, these p53 mutant mice did not develop early-stage lymphoma [176,177]. KRAS depletion and the depletion of RalB and IκB-related TANK-binding kinase 2 (TBK1) induced activation of p53 in a ROS- and NRF2-dependent manner. Similarly, the IκB kinase inhibitor BAY 11-7085 and dominant-negative mutant IκB-αM inhibited NF-κB activity and increased the levels of phosphorylated p53, p53, and p21^Cip1^ in a ROS-dependent manner [178]. The p53–Mdm2 protooncogene (MDM2)–ARF network can lead to unconventional and unique innovative approaches for developing a new generation of genetically informed and clinically effective cancer therapies [179].

Wild-type TP53 repressed Jdp2 promoter activity, but TP53-knockout mutant cells did not, which was demonstrated using HCT116 p53^−/−^ cells [180]. JDP2 enhances the NRF2-dependent antioxidant response [181]. JDP2 functions not only as a transcription factor but also as a histone chaperone for targeting specific acetylated histones. In addition, it has inhibitory effects on p300-mediated histone acetylation [182]. Therefore, the expression of TP53 and JDP2 was reported to be coregulated during transcription and chromatin regulation. These alterations might influence the function of NRF2 in TP53-mutant cancer cells or organoids. Thus, the molecular interactions of mutated TP53, JDP2, and NRF2 should be clarified.

Therefore, the contradictory results with PEA or CIN and SFN should be studied at the molecular level to identify the distinct pathways involved in human GC.

## 7. Discussion

**Figure 4:**
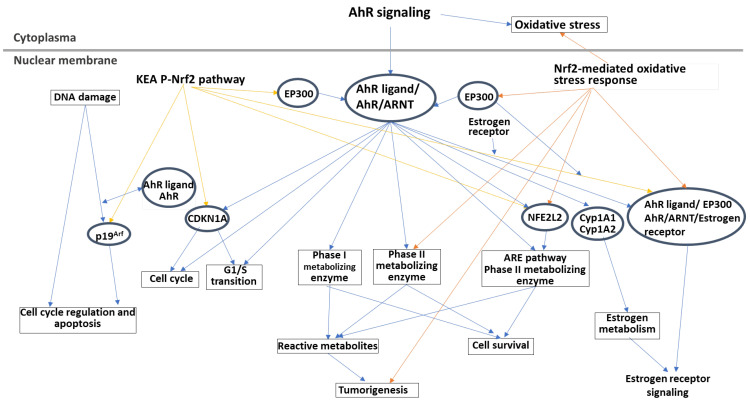
IPA analysis of the signaling networks between AhR signaling and NRF2 signaling. The canonical pathways of aryl hydrocarbon receptor (AhR) signaling (blue arrow), NRF2-mediated oxidative stress response pathway (orange arrow), and KEAP1-NEF2L2 (NRF2) pathway (yellow arrow) are selected from the Ingenuity Pathway Analysis (IPA) system (https://www.nihlibrary.nih.gov/resources/tools/ingenuity-pathways-analysis-ipa accessed on 30 August 2024). For the construction of signaling networks, AhR signaling is considered as the major axis, and the other two pathways are indicated with “CP: KEAP1-NEF2L2 Pathway” and “CP: NRF2-mediated Oxidative Stress Response”, respectively. The web-based IPA bioinformatic software is applied to overlay with each signaling pathway and to indicate the crosstalk molecules by the cross line between two signaling pathways.

This review highlights the role of the transcriptional balance of key factors between cellular antioxidation and oxidation in cancer development. The dedicated interplay to control NRF2 and AHR master regulators of ROS homeostasis should be defined as the context-dependent nature of NRF2/AHR actions in each cancer case. Ingenuity pathway analysis (IPA) of the signal networks between AHR and NRF2 revealed that they connected to shared target genes, such as genes related to the cell cycle, and chromatin regulators, including cyclin-dependent kinase inhibitor 1 (CDKN1A; p21^CIP1^) and EP300 (histone acetylase p300) (Figure 4). JDP2, such as CDKN1A, has been reported to play a role in cell cycle regulation by controlling cell growth via cyclin A2 [183] and p300/CBP acetyltransferase to function as a HAT on histones [182].

Given the complexity of antioxidation mechanisms in different cancer patients, the cancer types, and stages of cancer progression, manipulating redox status may be inappropriate. Thus, the precise regulation of redox balance is important and has become a key target in searching for the next generation of redox-regulating agents for cancer therapy. Repeating the examples to perform experimental validation would provide a further understanding of redox mechanisms in cancer initiation and progression.

A variety of responses to NRF2-mediated cancer development, which are obtained by 2-D cell culture, should be reexamined using 3-D organoids. These responses might be due to the intratumor heterogeneity of CSCs and to committed subpopulations with distinct microenvironments. The following questions should be addressed: (i) Do antioxidant exposures, as cancer treatments, demonstrate the positive and negative dual effects in cancer types, cancer stages or an endogenous ROS-dependent manner? (ii) Are there specific treatments with different doses in different tumor tissues that need treatment with antioxidant therapies for the required clinical outcomes? (iii) Is JDP2–NRF2-induced metabolic reprogramming in response to phase II enzyme ligands required for CSCs or niches that are dependent upon the status of TP53 mutation? Such questions are currently ongoing. The techniques used to measure endogenous ROS levels in vivo and in 3-D organoid tips might be the next targets for applying redox drug therapy for cancer treatment, in addition to further identifying markers for CSCs and microenvironments. These technologies might validate antioxidant therapy for tumors. Thus, we need more experiments involving mechanistic analysis of phase II drugs against cancer development.

Many preclinical trials of phase I and II agonists and antagonists have been conducted to date; however, only limited clinical trials have been conducted to evaluate the efficacy of antioxidative stress agents and antioxidants. The clinical and preclinical trials of phase I and II enzyme agents were performed to examine their efficacies for disease and cancer. However, none of them produced satisfactory outcomes in cancer therapy.

We have listed current applications [124,148,149,150,151]. *N*-acetylcysteine has been applied in various diseases, including cancers, and pulmonary, renal, liver (non-alcoholic fatty liver) diseases, and infections, including with human immunodeficiency virus, obesity, Parkinson’s disease, and depressive disorders. Melatonin has also been used in many diseases, including necrotizing enterocolitis, multiple sclerosis, and septic shock. Curcumin has been tested for a wide variety of conditions, including renal transplantation disorder, coronary artery disease, metabolic syndrome, and kidney disease (Table 2). These data were collected from the website http://clinicaltrials.gov (accessed on 28 August 2024) using the keywords antioxidative stress, antioxidation, diseases, drug treatments, and nutrients, and included ongoing and completed clinical trials.

## 8. Conclusions

Antioxidant drugs targeting phase II enzyme pathways have been extensively developed to focus on controlling ROS balance in patients with disease or cancer. The phase II enzyme regulating transcription factor NRF2 plays a critical role in modulating cancer development against oxidative stresses and the somatic mutations of genes and epigenetics. More than 600 genes, about 200 of which encode cytoprotective proteins, are involved in disease and cancer. In conclusion, modulating NRF2 activity is a promising approach to achieving homeostasis during every stage of tumorigenesis, where oxidative stress is an essential player. Nevertheless, a crucial strategy for cancer therapy is a better understanding of how activation or inhibition of NRF2-AHR pathways and ROS control functions. The cell specificity, the stage of cancer development, and the expression of genes encoding TP53, p21^Cip1^, NRF2, and JDP2 should be clarified. In addition, whether antioxidant drugs could affect NRF2 expression only or the *AHR*–*NRF2*–*JDP2* battery is also a critical issue. Thus, a deeper understanding of the role of antioxidants in cancer is required for developing new drugs [17,151,184]. The targeted therapeutics should be developed carefully to focus on ROS balance in each tumor type, cancer stem cells, and their stem cell niches.

## Figures and Tables

**Table 2:** Current summary of preclinical and clinical trials of AHR and NRF2 targeted drugs against cancers and diseases.

Drugs	Mode of Function	Diseases & Cancer	Clinical Trial	Performance
**[AHR Target]**				
IK-175	Inhibitor	Urothelial Cancer Advanced solid tumor	NCT104200963	[34]
BAY-2416964	Antagonist	Head neck cancer Lung cancer Colon cancer	NCT04069026	[119]
			NCT04069036	[119]
Sorafenib	Antagonist	Liver cancer Ovarian cancer Renal cell cancer		[120,121,122]
AFP464 (Amino flavone)	Antagonist	Solid tumors		
5F203 (Benzothiazole)	Antagonist	Renal Cancer		
JTE061 Tapinarof	Agonist	Atopic dermatitis Plaque psoriasis	NCT05680740	[115,123]
			NCT05142774	[115,123]
**[NRF2 Target]**				
Omaveloxolone	Antagonist	The first FDA-approved drug for Friedreich’s ataxia (FA)	NCT02255436	[124]
N-acethycysteine	Agonist	Melanoma	NCT01612221	[125]
		Leukemia	NCT05611086	[126]
		SARS-CoV-2-infection	NCT04792021	[127]
		HIV infection	NCT01962961	[128]
Melatonin	Agonist	Necrotizing enterocolitis	NCT05033639	[129]
		Multiple sclerosis	NCT02463318	
Melatonin + Vitamin C and E, N-Acethycysteine	Agonist	Septic shock	NCT03557229	[130]
Curcumin paclitaxel	Agonist	Chemotherapy peripheral neuropathy	NCT05966441	
Luteolin	Inhibitor	Tongue carcinoma	NCT03288298	[131]
Halofuginone	Inhibitor	Unspecified adult solid tumor	NCT00027677	[132]
		AIDS-related Kaposi sarcoma	NCT00064142	[132]
Berberine	Inhibitor	Colorectal adenoma	NCT03281096	
		G-R nonsmall lung adenocarcinoma	NCT03486496	[133]
		Gastric Cancer (Heliobacter pylori)	NCT04697186	[134]
		Gastric Cancer (Heliobacter pylori)	NCT03609892	[135]
		Colorectal adenoma	NCT02226185	[136]
		Colorectal adenoma	NCT03333265	[137]
Ascorbic acid	Inhibitor	Breast Cancer	NCT03175341	
		Bladder Cancer	NCT04046094	[138]
Ascorbic acid + mFOL FOX6	Inhibitor	Gastric Cancer	NCT03015675	
Vitamin C	Inhibitor	Prostatic neoplasm	NCT01080352	[139,140]
Gemcitabine + Ascorbic acid	Inhibitor	Pancreatic neoplasm	NCT01049880	[141,142]
Vitamin C + Supplement	Inhibitor	Non-small cell lung cancer	NCT02655913	[143]
Ascorbic acid + Sorafenib	Inhibitor	Metastatic Hepatocarcinoma	NCT01754987	[144]
Vitamin C, E and Zinc	Inhibitor	Skin neoplasms	NCT02248584	[145,146]
Biopsy, Osimextinb Triptolide analog	Inhibitor	Advanced lung non small cell carcinoma	NCT05166616	
Minnelide^TM^ capules	Inhibitor	Gastric, breast, pancreatic, prostate colorectal, stomach cancer	NCT03129139	
Minnelide	Inhibitor	Adeno squamous II carcinoma of the pancreas	NCT04896073	[147]
		Pancreatic Cancer	NCT03117920	

## Abbreviations

AhR	aryl hydrocarbon receptor
AP-1	activation protein-1
ARE	antioxidant response element
BAX	Bcl-2-associated X protein
BCL2	B-cell lymphoma 2
BCRP	breast cancer-resistant protein
BRCA1	breast cancer susceptibility gene 1
CBP	CREB binding protein
CDKN1A	cyclin-dependent kinase inhibitor 1
CE	cinnamaldehyde
COX2	cyclooxygenase 2
CRC	colorectal cancer
CREB	cyclic AMP response element binding protein
CSCs	cancer stem cells
DPx2	glutathione peroxidase 2
DSS	dextran sodium sulfate
EMT	epithelial-to-mesenchymal transition
ERBB2	Erb-B2 receptor tyrosine kinase 2
GC	gastric cancer
GLUTs	glucose transporters
G6PD	glucose-6-phosphate dehydrogenase
GPR78	glucose-regulated protein 78
GSH	glutathione-SH
GSK3	glycogen synthetase kinase 3
GSSG	glutathione sulfide
H_2_O_2_	hydrogen peroxide
HIF-1β	hypoxia inducible factor beta
HNSCC	head and neck squamous cell carcinoma
HO-1	heme oxygenase 1
3-IAld	indole-3-aldehyde
ICI	immune checkpoint inhibitor
IKK-α:-β	inhibitor of nuclear factor κB kinase subunit-α,-β
IPA	ingenuity pathway analysis
iNOS	nitric oxide synthase
JDP2	Jun dimerization protein 2
JNKs	c-Jun N-terminal kinases
Keap1	Kelch-like ECH associated protein 1
LPS	lipopolysaccharide
MARCO	macrophage receptor with collagenous structure
MDR1	multidrug resistance protein 1
MRP1-5	multidrug resistance-associated protein 1-5
MSCs	mesenchymal stem cells
NAC	*N*-acetylcysteine
NFkB	nuclear factor kappa B
NO	nitrogen oxide
NQO1	NAD(P)H: quinone dehydrogenase 1
NRF2	nuclear factor erythroid 2-related factor 2
NSCLC	non-small cell lung cancer
OXPHOS	oxidative phosphorylation
PEA	perillaldehyde
PGD	phosphoglucomutase dehydrogenase
PI3K	phosphoinositide 3-kinase
p-PERK	phosphorylated protein kinase RNA-like ER kinase
PSMA2	proteasome 20S alpha 2
PSMC4	26S proteasome regulatory subunit 4
ROS	reactive oxygen species
SFN	sulforaphane
SOD	superoxide dismutase
SQSRM1	sequestosome-1
TALDO1	trans-aldolase
TBK1	TANK-binding kinase 2
TKT	transketolase
TNF	tumor necrosis factor
β-TrCP	β-transduction repeat-containing E3 ubiquitin-protein ligase
Trx	thioredoxin
TrxR1	thioredoxin reductase 1
VEGF	vascular endothelial growth factor
x-CT	SLC7a11 cystine/glutamate transporter
